# Evaluating the Acceptability of the Drink Less App and the National Health Service Alcohol Advice Web Page: Qualitative Interview Process Evaluation

**DOI:** 10.2196/42319

**Published:** 2024-07-18

**Authors:** Melissa Oldham, Larisa-Maria Dina, Gemma Loebenberg, Olga Perski, Jamie Brown, Colin Angus, Emma Beard, Robyn Burton, Matt Field, Felix Greaves, Matthew Hickman, Eileen Kaner, Susan Michie, Marcus R Munafò, Elena Pizzo, Claire Garnett

**Affiliations:** 1 Behavioural Science and Health University College London London United Kingdom; 2 Herbert Wertheim School of Public Health and Human Longevity Science University of California San Diego San Diego, CA United States; 3 School of Health and Related Research University of Sheffield Sheffield United Kingdom; 4 Addictions Directorate Office for Health Improvement and Disparities London United Kingdom; 5 Institute of Psychiatry, Psychology and Neuroscience Kings College London United Kingdom; 6 Department of Psychology University of Sheffield Sheffield United Kingdom; 7 Department of Primary Care and Public Health Imperial College London London United Kingdom; 8 National Institute for Health and Care Excellence London United Kingdom; 9 Population Health Sciences, Bristol Medical School University of Bristol Bristol United Kingdom; 10 Population Health Sciences Institute Newcastle University Newcastle upon Tyne United Kingdom; 11 Centre for Behaviour Change University College London London United Kingdom; 12 School of Psychological Science University of Bristol Bristol United Kingdom; 13 Medical Research Council Integrative Epidemiology Unit University of Bristol Bristol United Kingdom; 14 Department of Applied Health Research University College London London United Kingdom

**Keywords:** alcohol reduction, digital intervention, acceptability, mobile health, mHealth, mobile phone

## Abstract

**Background:**

The extent to which interventions are perceived as acceptable to users impacts engagement and efficacy.

**Objective:**

In this study, we evaluated the acceptability of (1) the smartphone app Drink Less (intervention) and (2) the National Health Service (NHS) alcohol advice web page (usual digital care and comparator) among adult drinkers in the United Kingdom participating in a randomized controlled trial evaluating the effectiveness of the Drink Less app.

**Methods:**

A subsample of 26 increasing- and higher-risk drinkers (Alcohol Use Disorders Identification Test score≥8) assigned to the intervention group (Drink Less; n=14, 54%; female: n=10, 71%; age: 22-72 years; White: n=9, 64%) or usual digital care group (NHS alcohol advice web page; n=12, 46%; female: n=5, 42%; age: 23-68 years: White: n=9, 75%) took part in semistructured interviews. The interview questions were mapped on to the 7 facets of acceptability according to the Theoretical Framework of Acceptability: *affective attitude*, *burden*, *perceived effectiveness*, *ethicality*, *intervention coherence*, *opportunity costs*, and *self-efficacy*. Alongside these constructs, we also included a question on *perceived personal relevance*, which previous research has linked to acceptability and engagement. Framework and thematic analysis of data was undertaken.

**Results:**

The Drink Less app was perceived as being *ethical*, *easy*, *user-friendly*, and *effective* for the period the app was used. Participants reported particularly liking the tracking and feedback sections of the app, which they reported increased *personal relevance* and which resulted in positive *affect* when achieving their goals. They reported no *opportunity cost*. Factors such as negative *affect* when not meeting goals and boredom led to disengagement in the longer term for some participants. The NHS alcohol advice web page was rated as being *easy* and *user-friendly* with no *opportunity costs*. However, the information presented was not perceived as being *personally relevant* or *effective* in changing drinking behavior. Most participants reported neutral or negative *affect*, most participants thought the alcohol advice web page was accessible, and some participants reported *ethical concerns* around the availability of suggested resources. Some participants reported that it had acted as a starting point or a signpost to other resources. Participants in both groups discussed motivation to change and contextual factors such as COVID-19 lockdowns, which influenced their perceived *self-efficacy* regardless of their assigned intervention.

**Conclusions:**

Drink Less appears to be an acceptable digital intervention among the recruited sample. The NHS alcohol advice web page was generally considered unacceptable as a stand-alone intervention among the recruited sample, although it may signpost and help people access other resources and interventions.

## Introduction

### Background

Drinking alcohol at increasing- and higher-risk levels is a major public health concern and contributes to health inequalities, with the most deprived groups experiencing the most harm from alcohol [1]. Fewer than 7% of increasing- and higher-risk drinkers who visited their general practitioner in the last year received face-to-face interventions in primary care to support alcohol reduction [2]. Key barriers to the delivery of these interventions by practitioners are lack of time, low confidence about discussing alcohol with patients, and lack of training [3-5]. Digital interventions, such as websites, are effective for reducing alcohol consumption compared with no intervention or minimal input controls [6]. They may overcome delivery barriers, as they potentially have a broad reach and relatively low implementation costs (once developed), so they can be delivered at scale [7]. Smartphone apps are a promising type of digital intervention, as smartphones have become increasingly affordable to end users and prevalent among the UK population [8]. However, despite the availability of hundreds of alcohol-related apps on commercial app stores, the majority have been developed without reference to scientific evidence or theory [9]. Furthermore, few have undergone evaluation in terms of their acceptability, engagement, or effectiveness [6]. It is critical to establish whether digital interventions are acceptable to end users, as acceptability impacts engagement and effectiveness [10]. Furthermore, demonstrating the acceptability of interventions can encourage public health practitioners and policy makers to promote interventions to those who would benefit from using them.

The extent to which interventions are perceived as acceptable to users and to other stakeholders such as family members, health care professionals, and policy makers affects engagement and effectiveness [10]. Acceptability sits at the core of the Technology Acceptance Model [11], which states that the perceived ease of use and perceived usefulness of a given technology positively influence use intentions. Most definitions of acceptability in digital health research primarily capture how people think and feel about a given technology [12,13], an example being “an emergent property, or a ‘gut feeling’, arising from a dynamic, complex system of emotional and cognitive components” [14]. The Theoretical Framework of Acceptability (TFA) defines acceptability as a multifaceted construct reflecting the extent to which a health care intervention is considered appropriate based on anticipated or experienced emotional and cognitive responses to the intervention [12]. Acceptability, according to the TFA, consists of 7 facets: affective attitude, burden, perceived effectiveness, ethicality, intervention coherence, opportunity costs, and self-efficacy. In addition to the facets outlined by the TFA, the extent to which an intervention is perceived as being personally relevant and tailored to the individual could be of importance when thinking about acceptability [15] and has shown to be linked to engagement [16].

### This Study

Drink Less is a theory- and evidence-informed app-based intervention designed by researchers [17] to help people reduce their alcohol consumption. This study examined the acceptability of Drink Less and the National Health Service (NHS) alcohol advice web page [18] to adults drinking at increasing- and higher-risk levels in the United Kingdom following their participation in a randomized controlled trial evaluating the effectiveness of the Drink Less app [19] compared with usual digital care (eg, NHS alcohol advice web page [18]). It aimed to assess participants’ views on the acceptability of the Drink Less smartphone app and of the NHS alcohol advice web page.

## Methods

### Ethical Considerations

Ethics approval was obtained from University College London (UCL) Research Ethics Committee (16799/001). All participants provided informed consent before participating in the study. Data were anonymized and securely stored. All study participants were renumerated with a GBP £20 (US $25.46) Amazon (Amazon Inc) voucher to thank them for their time.

### The Drink Less App

The development of Drink Less was informed by research findings and behavioral theories such as the Capability Opportunity Motivation–Behavior model of behavior change [17]. Drink Less consists of evidence-based modules to help users change their drinking behavior: Goal Setting (setting weekly drinking reduction goals), Self-Monitoring and Feedback (monitoring alcohol consumption and seeing progress on goals), Action Planning (creating plans for dealing with difficult drinking situations), Normative Feedback (providing personalized feedback on how an individual’s drinking behavior compares with the norm), Cognitive Bias Re-Training (a game for retraining users’ automatic biases for alcoholic drinks), Insights (providing users with weekly and monthly feedback on alcohol consumed), Behavioural Substitution (planning to substitute drinking with a neutral behavior), and Information about Antecedents (providing users with information about situations, events, emotions, and cognitions that predict their drinking [20]).

### NHS Alcohol Advice Web Page

The web page is freely accessible and appears in the top Google searches for “alcohol reduction advice” and “how to drink less alcohol” in the United Kingdom. The web page contains tips for cutting down on alcohol consumption, such as planning, setting a budget, and switching to smaller or weaker strength drinks. This is presented alongside benefits of cutting down for physical and mental health, including weight loss and improvements in mood and sleep [18]. The web page also has links to other web pages, including “alcohol support” and “the risks of drinking too much.”

### Study Data

This study analyzed data collected within the iDEAS (iOS Drink Less, evaluating the Effectiveness of an Alcohol Smartphone app) trial, a large-scale randomized controlled trial [19] evaluating the effectiveness and cost-effectiveness of recommending the Drink Less app compared with usual digital care (the NHS alcohol advice web page) in the United Kingdom using an embedded mixed methods process evaluation. This paper reports the analysis of the qualitative interviews assessing the acceptability of the interventions.

Participants were eligible for the iDEAS trial if they were aged ≥18 years, lived in the United Kingdom, were increasing- and higher-risk drinkers (Alcohol Use Disorders Identification Test score≥8), had access to an iOS (Apple Inc) device (ie, iPhone, iPod touch, or iPad), and wanted to drink less alcohol. Recruitment ran from July 2020 to March 2022, with the final follow-up conducted in October 2022. Recruitment occurred via a multipronged strategy, including an advertisement on the NHS website, targeted and untargeted social media advertising, radio advertising, a mail-out to a database of UK-based users of the Smoke Free app, and local advertising through health care providers.

When participants signed up for the iDEAS trial, they provided informed consent to participate in 3 web-based follow-up surveys after 1, 3, and 6 months. They were given the option to also consent to be contacted for a follow-up interview. Participants were then asked again at their 6-month follow-up whether they would be happy to be contacted for a follow-up interview about their experience of using the intervention.

### Participants

Participants were selected from the group who consented at either baseline or 6-month follow-up to an interview. We identified participants purposively to interview roughly equal groups of men and women, those on low and high incomes, and from a range of ages and ethnic backgrounds. This was to ensure that the views of a diverse group of participants were represented. Researchers also purposively sampled to include people with a range of app engagement levels to avoid recruiting only highly engaged participants who may have felt more positively about the intervention. For those in the Drink Less group, engagement data were used to determine whether they had low (defined as 1-2 recorded sessions), medium (3-27 sessions), or high (≥28 sessions) engagement with the app. Participants were asked whether they used the intervention in their 1- and 6-month follow-up surveys, and these data were used to ensure that different levels of engagement within the comparator group (eg, never used vs used) were captured. When sampling, researchers filtered the data based on demographic and engagement criteria, and the first person meeting the required criteria was invited. To include the views of those who may have been less engaged in the study, those who provided consent to participate in the interview at baseline but did not complete the 6-month follow-up were invited to participate.

The final sample was 26 participants (Drink Less group: n=14, 54%; NHS alcohol advice web page [comparator] group: n=12, 46%). Recruitment ceased when meaning saturation was reached, and no further nuances or insights were found [21]. No new codes were identified in later interviews that differed from those identified in the earlier interviews. Nor did the later interviews change the meaning of any codes or themes.

### Epistemological Position

We adopted a realist epistemology, assuming that our interpretation of each participant’s reality is shaped through their perception of that reality. The realist epistemology assumes that meaning and experience are reflected in language [22]. This epistemology fitted our research aim of exploring acceptability judgments for the 2 interventions.

### Procedure

In line with previous conceptualizations of acceptability [14], the interview topic guide (Multimedia Appendix 1) was designed to first measure an individual’s gut feeling about the intervention they received (using a 5-star rating system) before exploring the 7 component facets according to the TFA [12], namely affective attitude, burden, perceived effectiveness, ethicality, intervention coherence, opportunity costs, and self-efficacy and perceived personal relevance [16]. Given that trial architecture (eg, randomization and follow-up) can be conflated with perceptions of the acceptability of the studied intervention [23], we asked participants to reflect on each separately.

We consulted a public and patient involvement group to ensure that the questions were clear for participants to answer. Public and patient involvement feedback was that the ethicality question “How fair did you think the intervention was to all possible users?” would be difficult to answer, and the suggested alternative wording was “Do you think anyone could use this intervention?” Participants also suggested that the burden question that originally read, “How difficult did you find it to use the intervention?” be split into 2 questions focusing on how time consuming the app was and whether there were any other difficulties. Both suggested changes were implemented.

Once selected, participants were emailed and invited to attend a one-to-one semistructured interview. Interviews were conducted within 2 months of participants finishing their final follow-up survey. A total of 24 interviews took place using videoconferencing software, and 2 interviews took place on the phone (participants’ preference). At the start of the interview, the researcher confirmed consent. The interview focused on perceptions of the acceptability of the interventions. The interview was led by the topic guide, exploring each facet of acceptability in turn. After each question, the interviewer prompted participants to expand on their answers or asked relevant follow-up questions to ensure that participants fully expressed their views. All interviewers conducted at least 1 pilot interview. Interviews were recorded using a Dictaphone. They took between 12 and 34 minutes to complete. Multimedia Appendix 2 contains the COREQ (Consolidated Criteria for Reporting Qualitative Research) checklist [24].

### Research Team and Reflexivity

#### Personal Characteristics of Interviewers

There were 3 interviewers. MO is a female senior research fellow at UCL and has a PhD in health psychology. She is predominantly a quantitative researcher but undertook qualitative interviewing training in advance of the interviews. LMD is a female research assistant at UCL and has an MSc in psychiatric research. She has experience in conducting interviews remotely and has undertaken training sessions in qualitative research. GL is a female research fellow (trial manager) at UCL and has an MSc in developmental and educational psychology. She has experience undertaking qualitative interviews and conducting interviews remotely by phone.

#### Relationship With Participants and to the Topic

All interviewers had little contact with participants before the interview, save for reminder emails sent at 1, 3, and 6 months. Interviewers may potentially also have made up to 2 reminder phone calls and sent a postcard and a letter at the 6-month follow-up. Participants may have had some knowledge of the interviewers and their roles within UCL and the trial team through study documentation. The goal of the research, to understand the acceptability of the digital intervention used, was explained to participants at the start of the interview. All 3 interviewers were blind to the outcome data at the time of the interviews.

#### Reflections on the Interview Process

The 3 interviewers thought that their demographic characteristics did not seem to impact the interview process. Participants seemed able to speak openly about their positive and negative experiences of using the app and web page. However, there are 2 points of interest. First, when participants reported negative points about the NHS alcohol advice web page, they often prefaced these comments by talking very positively about the NHS more generally. Second, in both groups, some participants apologized to the interviewer before reporting a negative experience or saying something they did not like about the web page or app. These points could suggest that participants experienced some social desirability bias and thought that the interviewers wanted them to report positive experiences.

### Analysis

Interviews were transcribed verbatim, anonymized, and then uploaded into NVivo (version 12; QSR International) for coding and analysis. We followed a combined inductive and deductive approach. An initial coding framework was developed using a priori themes (eg, TFA facets and perceived personal relevance). Two researchers (MO and LMD) coded the first 5 interviews separately, and then an iterative process of cross-checking coding strategies and data interpretation was carried out to establish a consensus and develop a revised coding frame. Coding was further refined using an ongoing comparative method, whereby each interpretation and finding was compared with existing findings, as more transcripts were analyzed. Following initial coding, similar responses within each construct were inductively analyzed to generate content themes [22] representing how that construct contributed to the reported acceptability. Participant quotes are presented alongside the age and sex of the participants. Where duplicated, a letter has been added to the age.

Following analysis, a draft of the *Results* section of this report was shared with a subsample of 8 randomly selected participants (4 from each group). Participants were asked to comment on how well the relevant *Results* section summarized their views on the acceptability of the digital tool and whether there were key points that they thought were missing from this summary. Participants largely thought that the *Results* accurately represented their experiences, and no changes were made. Feedback is presented in Multimedia Appendix 3.

## Results

### Participant Characteristics

Of the 5602 trial participants, 4443 (79.31%) consented to the interview at baseline or 6-month follow-up. A total of 55 people were invited to the interview, of whom 53% (n=29) declined or did not respond, resulting in a final sample of 26 participants. Table 1 provides an overview of participant characteristics overall and by group.

A definition and an overview of themes are presented in Table 2 for both groups.

**Table 1:** Participant characteristics overall and by group.

			Drink Less (n=14)		NHS^a^ alcohol advice web page (n=12)		All (n=26)
Sex (female), n (%)			10 (71)		5 (42)		15 (58)
Age (y), mean (SD; range)			41.64 (16.19; 22-72)		43.50 (14.08; 23-68)		42.50 (14.98; 22-72)
**Ethnicity, n (%)**							
	Asian	1 (7)		0 (0)		1 (4)	
	Black	1 (7)		1 (8)		2 (8)	
	White	9 (64)		9 (75)		18 (69)	
	Mixed race	2 (14)		1 (8)		3 (12)	
	Other	1 (7)		1 (8)		2 (8)	
Higher income^b^, n (%)			10 (71)		8 (67)		18 (69)
**Engagement, n (%)**							
	Low	2 (14)^c^		—^d^		—	
	Moderate	5 (36)		—		—	
	High	7 (50)		—		—	
	Ever used	—		10 (83)		—	
	Not used^e^	—		2 (17)^e^		—	
Global acceptability, mean (SD; range^f^)			3.86 (0.74; 2-5)		2.72 (1.20; 1-5)		—

^a^NHS: National Health Service.

^b^Earning more than £26,000 (US $33,093.58).

^c^These participants were originally categorized as nonusers of the app based on engagement data but self-reported briefly using the app in interviews; they may not have provided accurate linking data between the app and trial so they are categorized as having low engagement here.

^d^Not applicable.

^e^Reported never using the National Health Service (NHS) alcohol advice web page at 6 months but reported looking at it briefly at the start of the trial in the interviews.

^f^Participants were asked to judge the global acceptability of the intervention on a scale of 1 to 5 stars.

**Table 2:** Definition and overview of themes.

Theme	Definition of facet	Drink Less app	NHS^a^ alcohol advice web page
Affective attitude	How an individual feels about the intervention	Generally liked the app, particularly the drinking diary Felt proud and happy when meeting goals Some negative affect when failing to meet goals	Most reported neutral or negative affect Some felt that the web page was patronizing
Burden	The perceived amount of effort necessary to use intervention	Quick and easy to use Could tailor time spent based on time available or support required Repetitive in the longer term	Quick and easy to use Some framed this as a negative aspect and thought the web page was too basic
Ethicality	The extent to which the intervention has a good fit with an individual’s value system	Generally accessible Mixed views on whether the app would work for those who had less experience of digital tools	Generally accessible Concerns around the availability of treatment through the NHS as recommended on the web page and confidentiality of revealing drinking to health care professionals
Intervention coherence	The extent to which the participant understands the intervention and how it works	The app was generally considered intuitive Some reported a learning curve or difficulties with features Participants reported understanding many of the mechanisms of change (eg, tracking and goal setting)	Easy to use and navigate
Opportunity costs	The extent to which benefits, profits, or values must be given up to engage with the intervention	Most reported no opportunity costs Some thought that reducing drinking impacted social life Others reported reducing lone drinking rather than social drinking, which they felt would have been more of an opportunity cost	No opportunity costs
Perceived effectiveness	The extent to which the intervention is perceived as likely to achieve its aim	Most thought the app helped them reduce their drinking for the time that they used it Some contextual factors were a barrier to change (eg, the COVID-19 pandemic) Disengagement from the app in the longer term	Most thought that the web page did not directly help them reduce their drinking Some found it helpful as a signposting tool to other resources
Perceived personal relevance^b^	The extent to which the intervention is suited to the participant’s individual needs	Highlighted the tailored toolbox nature of the tool—could pick the features they liked Remote nature did not suit some, but some liked the anonymity	Many thought they already knew the information, and it was too generic Participants reported that the web page might be helpful for other people but was not personally suited to them
Self-efficacy	The participant’s confidence that they can perform the behaviors required to participate in the intervention	Mixed confidence in whether the app would work for them Trust and confidence associated with UCL^c^ branding Importance of personal motivation	Mixed confidence in whether the web page would work for them Importance of personal motivation

^a^NHS: National Health Service.

^b^Not a facet of the Theoretical Framework of Acceptability.

^c^UCL: University College London.

### The Drink Less App

#### Affective Attitude

Participants generally liked the Drink Less app. They reported particularly liking the tracking component (Self-Monitoring and Feedback) and the traffic light color–coded feedback on the calendar for alcohol-free, light, and heavy drinking days:

I quite liked the little graph that shows whether you’re on track.

Female, 60 years

I liked the fact that it was nice and visual so the calendar, where it came up with red, green or amber I found that quite useful for me.

Female, 38a years

Participants reported feeling positive and proud when they were meeting their goals to reduce their consumption:

I was encouraged by the calendar where you have the days when you don’t drink, and having it consistently green week after week, it made me feel nice and I wanted to continue doing that.

Male, 41 years

I did like the fact that if I logged on that I hadn’t drank it sort of praised me.

Female, 55 years

A few participants reported negative affect when recording heavy drinking, relapsing, or failing to meet their goals. They reported not liking having a visual account of their failure:

I didn’t want to visually see my mistakes on my phone and yeah I think judgment on myself as well.

Female 26A years

When you do start going backwards and drinking more and more than you know you’re all you’re doing is sort of putting effectively negative data into the app. And you just feel like you’ve let yourself down, you’ve let the app down and you’ve let your progress down.

Male, 22 years

#### Burden

Participants generally reported that the app was not time consuming to use and that they found it quite easy and user-friendly:

It is easy to use, it’s quick it’s not onerous. (Female, 47 years)

Some participants talked about spending more or less time on the app based on the level of support they felt they needed each day or the amount of time they had available:

I could spend over an hour going through it and making a plan and you know for that if I thought I needed the extra support and other days it just took five minutes, maybe 10 to just log in you know what was happening.

Female, 67 years

Other times I’m kind of free, so I don’t see maybe I’m not doing anything I’m just bored or something like that and I start using the app...so sometimes time consuming sometimes not.

Male, 30B years

In the longer term, using the app was described by some as repetitive. Over time, this led to negative emotion or forgetting and eventually disengagement:

As it went on I found it a bit of a chore and kind of forgot about it.

Female, 26A years

I was using it a lot at first. But then I sort of kind of lost interest in the app.

Male, 22 years

#### Ethicality

This theme is focused on fairness and accessibility. Participants reported thinking that the app was accessible for most people who had access to a smartphone:

Anyone who’s got a smartphone and uses Apps can use it, but that isn’t you know, obviously that isn’t everyone.

Female, 47 years

Participants felt that to be fair to all users, the app should be accessible to everybody. Some participants highlighted that those who are older or less technically able might struggle to use Drink Less. Others thought it was already broadly accessible:

I’m sure anybody can use it, but it needs to be a bit simplified if you want people who are less tech-savvy to use it.

Male, 41 years

I’m sort of thinking about my mom who isn’t very tech minded I think if the app was downloaded for her and she had a tablet... she’d still be able to use it as well, so yeah it’s easy to use for anybody I would think.

Female, 55 years

#### Intervention Coherence

Most said that the app was intuitive:

I didn’t have any problem with it, was very usable all the labels and options, they were all self-explanatory.

Male, 30A years

It was easy to download and easy to just get up, set up and start using.

Female, 55 years

Some participants reported a learning curve when they first started using the app, others reported specific issues with using different features of the app, such as logging cocktails for which there was no default option, entering the cost of drinks, and customizing goals:

In the beginning...I was a bit overwhelmed, what am I supposed to do now? Am I supposed to do this game or do that. But over time I realised that, first of all, you don’t have to do any of them, it will still work.

Male, 41 years

The one thing that I just found a bit confusing was like, if I had a drink and I didn’t know how to record it because yeah there wasn’t an option for some things.

Female, 25 years

Participants seemed to be aware of some of the underlying behavior change components through which the app worked. Participants commented on the importance of tracking in highlighting how much they drank and reported that the praise and the traffic light function in the calendar encouraged them to have more alcohol-free days. Others commented on reflecting on the effect that alcohol had on their mood and sleep and said the app dispelled myths around alcohol helping them sleep or relax:

I was encouraged by the calendar where you have the days when you don’t drink, and having it consistently green week after week.

Male, 41 years

Even if I wasn’t putting it into the app I was aware or reminded of the numbers of units per week that are heal- that’s healthy. And having you know, a glass just a glass of wine on a random night adds to that total and it’s unnecessary.

Female, 47 years

People always assume, or I’d always assumed, you have a drink and it helps you sleep but actually it really doesn’t so I’ve been able to just...think about that a little bit more.

Female, 38 years

#### Opportunity Costs

Most participants said the Drink Less app fit into their lifestyle and did not interfere with other obligations:

You know if I couldn’t do it that day, I could always try and do it the next day or anything like that or just not do it at all.

Female, 25 years

I’m really busy. I’ve got young children and a full time job and all the rest of it, and it was it was something that I have no problem incorporating into my routine.

Female, 38A years

Some participants suggested that the using the app to cut down on their drinking had indirect opportunity costs by interfering with their social life. Participants reported not wanting to drink more than their goal amount, and avoiding some social occasions with friends where they felt they might be tempted or pressured to drink:

I was like I only have four units left and like weekend is when I usually drink or like meet friends and things like that so yeah it was a it was a little bit like, I was kind of losing every week in a way.

Male, 30A years

Other people talked about how their use of the app did not impact their willlingness to socialize in drinking settings and how they used it more to cut out lone drinking or “mindless drinking”:

I think it made me more conscious of erm having the odd drink here and there...I can’t say it helped me with my drinking if I was out socializing in the pub.

Female, 55 years

If I’m going out with or with friends I will still drink, what it knocked on the head is the having a glass of wine while I’m cooking dinner for no particular reason drinking.

Female, 47 years

#### Perceived Effectiveness

Most participants thought the app helped them drink less for the time that they used it and described different strategies of using the app to successfully cut down their consumption. These included accurately monitoring units consumed with reference to the drinking guidelines, downsizing, cutting out habitual drinking, and aiming for more alcohol-free days:

Overall, I do think it helped me for that time, I think the other helpful thing was also just to like see in my life like what 14 units really looks like and how small It is.

Male, 30A years

I’ve not cut the days I drink back, but I instead of buying a whole bottle of wine, I buy a small bottle of wine.

Female, 72 years

When you see how many alcohol units you’re using per week, or you’re drinking per week it yeah it just kind of reset my drinking and it has knocked on the head the mindless drinking.

Female, 47 years

Not all participants thought the app helped them drink less. Some described contextual barriers to them drinking less, such as the COVID-19 pandemic and Christmas. Other participants described disengaging from the app and returning to heavier drinking in the longer term:

Perhaps, if I hadn’t started at Christmas maybe or if the situation would have been different, then maybe I would have taken more notice of it.

Female, 60 years

I used it religiously for a while and then and then after that it is hard once you drop off again like I said when you go backwards it’s hard to then go back on it and start again you just can’t be bothered like and frankly it’s like it’s really hard to find the energy to like start afresh.

Male, 22 years

Participants had some suggestions on how to make the app more effective for them in the longer term, which included changing the features of the app more frequently and using the app in group or health care settings:

Perhaps a weekly zoom meeting with 20 people that are using the app to see how people are getting on there’s no shame and you can sort of just say okay well okay you’ve you’ve taken a step back, but it’s fine input your data, the next day, and the next day, and this is how to actually come back.

Male, 22 years

#### Perceived Personal Relevance

Most participants reported that the app was a good fit for them. The app was commonly perceived like a toolbox with different intervention components available to select. People reported using the app in different ways and finding the components of the app that worked best for them. Some participants said that the remote nature of the app and the anonymity it offered were a good fit for them:

The good thing about it is that it has various tools and games and I am sure that not every one of these functions will appeal to every single user, so from my perspective, having a supermarket function was very useful.

Male, 41 years

I wasn’t in a position to go to the doctor and didn’t feel like seeing someone in person, so the app was a personal way of getting support anonymously.

Male, 41 years

Not everyone felt that the app was a good fit for them; some participants reported needing more support or not liking the digital and remote nature:

I don’t think that the just reading things on the screen was something that appealed to me, personally, but may appeal to others.

Female, 60 years

#### Perceived Self-Efficacy

Participants reported mixed levels of confidence in whether the app would work for them:

I was very confident it would work out for me.

Male, 30B years

I wasn’t particularly confident because I didn’t know what it was and how it would you know run run out and how I could use it and what other things were in it.

Female, 67 years

Participants reported having confidence and trust in the app based on its association with UCL:

I felt confident, I thought you know it’s by UCL, I am sure its trustworthy so yeah, I was confident.

Female, 25 years

Confident another way, I suppose I trusted it. I believe what it was telling me...I suppose I assumed by that that there’d been accurate research by the people that had developed the app.

Female, 55 years

Participants reflected on the importance of their own motivation to change their behavior and how this determined whether they were confident in the Drink Less app:

Now, can it help me now? I don’t think so because I don’t have the same motivation as in February when I started.

Male, 41 years

I think say someone I knew had a problem, and they were ready to really address it and sort it out I’d recommend it to them. Just to give it a go but. I just don’t think maybe I’m not there yet ready I don’t know, maybe in the future it be helpful.

Female, 26A years

### NHS Alcohol Advice Web Page

#### Affective Attitude

Participants generally reported neutral or negative affect when discussing the NHS alcohol advice web page. Some reported finding it basic and patronizing:

The website itself was fine it was okay I liked it no kind of bad feelings about the actual website or kind of irritations or anything.

Female, 40 years

More like er a nagging er nagging grandmother.

Male, 68 years

I found it vaguely informative, but a lot of it was just common sense, so in a way, in a way, some of it felt almost like a little bit patronising.

Male, 30 years

#### Burden

The NHS alcohol advice web page was generally considered very easy and quick to use:

Very easy to read and clear and concise.

Male, 30 years

However, this was also framed as a negative aspect of the web page. Some said they used the web page only briefly, as the information contained was basic. Others suggested that the lack of change or interactive features meant that they did not return to the web page:

Very, very sparse.

Male, 42 years

I mean you know it was only a few visits to it so um you know after that you you’re not going to gain any more from it.

Male, 68 years

#### Ethicality

Participants reported thinking that the NHS alcohol advice web page was accessible for most people who had access to the internet:

Anyone who can use internet could use the website.

Male, 42 years

I feel like it’s very accessible to all, especially in this day and age.

Male, 23 years

There was a range of concerns about the treatments offered by the NHS and around negative consequences if they honestly reported their alcohol consumption to a health care professional:

The help that they suggest is available isn’t always available easily.

Female, 55A years

I don’t think there’s enough done to help people with it [alcohol dependence] in all honesty I think it’s just a case of people get stuck in some sort of rehab which is generally lumped into some sort of mental welfare ward.

Male, 36 years

I was worried about driving lessons being taken away.. about social services, because I had a child things like that...if there was in the early days, some kind of reassurance that you could you know get a certain amount of help, without any repercussions then people would actually take part in the options that are available and suggested.

Female, 40 years

#### Intervention Coherence

Participants reported confidence in navigating the NHS alcohol advice web page; the titles were clear, and it was easy to use:

It was very clear and everything’s labelled nicely and kind of navigating the website is very, very simple.

Male, 23 years

I felt I could use it pretty instinctively.

Female, 26 years

#### Opportunity Costs

No participants reported that the NHS alcohol advice web page had interfered with anything else important in their life:

It wouldn’t be something that I’d look at instead of day to day life.

Male, 23 years

#### Perceived Effectiveness

Participants reported that while the NHS alcohol advice web page did not directly affect their drinking, they thought they would have benefited from more of a call to action:

It’s all good and well telling me all these facts and giving me information about how to deal with drinking and what it can do to you but really it’s not enough.

Male, 23 years

I sometimes wonder if they should be a bit harsher but you know like this is the consequences or something you know.

Female, 55A years

There was nothing in the NHS incentivised you to say oh right okay, but basically it was just basically textbook stuff on the internet.

Male, 68 years

For some participants, it served as a first step signposting to other resources or motivating them to independently search for other resources:

It started me on the journey and it pointed me in the right direction.

Female, 44 years

At least it made me look at other things, and eventually identify, something that was really helpful.

Female, 61 years

#### Perceived Personal Relevance

Many participants reported feeling like the NHS alcohol advice web page was not relevant to them. They thought that the information presented was too generic and would benefit from more tailored components:

For my my individual needs for what it was, I didn’t think it was that well suited to compared to like I said if there’d been something a little bit more tailored.

Male, 30 years

A kind of screening tool at that point might have been helpful to then identify people with moderate alcohol problems to severe alcohol problems. And then you could then guide them or direct them to a more appropriate channel rather than treat everybody the same whether they drink five bottles of spirits a day or two glasses of wine.

Female, 61 years

Many participants talked about the NHS alcohol advice web page perhaps being a good fit for other people and acknowledged the need for the web page to be quite generic given that its purpose was to serve a varied group of people:

It might have served other types of persons needs but not mine. Perhaps I wanted too much from it, perhaps I wanted something too individual.

Female, 61 years

It was good and informative but quite generic. You know, which is suppose it has to be if a lot of people are using it.

Female, 26 years

But because of what it is designed to be for the NHS I suppose it needs to be that way because it’ll have people who sort of drinking a bottle of vodka a day to someone who they thought I might give it a Google because, last night I hit it a little bit too hard.

Male, 30 years

#### Perceived Self-Efficacy

Participants reported mixed levels of confidence in using the NHS alcohol advice web page to reduce their drinking:

I don’t have a lot of faith in the NHS, as much as I love the NHS, I don’t have a lot of faith in the help that’s available for this type of thing.

Female, 55a years

It’s NHS so it’s recognisable and you feel comfortable using it, because it’s trusted source.

Male, 23 years

As in the Drink Less group, participants highlighted the importance of their own motivation to change:

I think that the study came at a really good time for me, that time that I was ready to face up to the fact that I needed help, and I wanted to be accountable as well.

Female, 44 years

## Discussion

### Principal Findings

Participants reported liking the Drink Less app; they particularly liked the tracking and feedback components, which they considered made the app more personally relevant to them, and felt proud and positive when they were meeting their goals. They discussed that the app functioned as a “supermarket” or toolbox, whereby they could choose and use the components of the app that worked best for them. Participants reported different strategies and goals, and most thought that the app was effective in reducing their alcohol consumption, particularly in the shorter term. This “supermarket” function extended to the depth of use, and participants also reported that use was influenced by the level of support they felt they needed that day—this could include spending more time on the app making plans and behavioral substitutions when their cravings were higher. Participants reported being confident in using the app and that it was intuitive and accessible. However, some thought that there was a learning curve at the start of using the app, and some participants reported specific difficulties in using the app, such as logging cocktails (which are not included as default options in the drinking diary) or customizing goals. Some participants reported negative affect when logging heavier drinking days or failing to achieve their goals, which led to disengagement in the longer term. Another factor reported as leading to disengagement was boredom. Some participants reported that although the app was not burdensome to use, it became something of a chore.

Participants reported that the NHS alcohol advice web page was very quick, easy, and intuitive to use and accessible to anyone with internet access. Participants reported that the web page could be a useful tool for other people, but they judged that the information contained was less personally relevant to them, and the web page was perceived as basic and generic. Some found that the web page had provided the starting point for them in reducing their alcohol consumption by signposting them to other tools or resources, whereas others thought it had not had an impact on their alcohol consumption. There were concerns raised by participants about contacting a health care professional, as some thought this would be ineffective and were not confident that the services would be available and one parent feared that social services would be contacted. Participants suggested that the web page could benefit from having some more personalized features, such as signposting for different levels of consumption.

We have avoided drawing comparisons between the 2 tools throughout this analysis because the nature of the 2 tools is different. The trial may have set up the evaluation of the acceptability of the tools to encourage participants to think of the NHS alcohol advice web page as a stand-alone or prolonged intervention. However, unlike the Drink Less app, the alcohol advice web page focuses on information provision and does not offer opportunities for personalization or engagement.

These data are consistent with work highlighting the importance of perceived personal relevance [15,16] and a recent evaluation of an app designed to help ex-service personnel reduce their drinking that highlighted credibility, ease of use, and personalization as important functions of the app [25].

Although participants perceived the app as potentially effective in helping them reduce their alcohol consumption in the shorter term, they felt that some factors, such as repetition and negative affect, led to their disengagement from the app in the longer term. Achieving prolonged engagement with digital interventions [26] and apps more widely [27] is a known challenge. Participants had some ideas, such as new features or group use, that could have boosted their engagement in the longer term. These findings suggest that the Drink Less app should be viewed as a dynamic intervention that new evidence-informed components and features could be added to. This does raise practical issues, as the app is currently managed by an academic research team with limited resources available for ongoing management and development costs. One implication of this research is, therefore, to consider how academics and third-sector partners can work together to continue to develop and maintain digital interventions with evidence of effectiveness and acceptability to end users.

Among this sample, Drink Less seems to be an acceptable intervention. However, the generalizability of these findings should be considered. Digital exclusion [28] is an important factor to consider when promoting digital interventions. Some people may be unable to access devices or data or be unable to make the most of them due to a lack of knowledge or resources or are less likely to engage with them [28]. Digital exclusion is more likely in vulnerable populations, including older people, those out of work, the most financially vulnerable people, and those who live with a condition that limits or impairs their use of communication services. Therefore, researchers and policy makers should be aware of how the introduction of digital interventions could impact widening inequalities, as digital interventions such as smartphone apps are not going to be accessible and acceptable for everyone. Metrics of variation across different protected characteristics should be measured in the roll out of all digital public health tools and explored in process work.

The topic guide was developed in collaboration with experts by experience to ensure the questions were understandable and addressed the underlying concepts. We asked participants to provide feedback on the *Results* sections (Multimedia Appendix 3), and participant feedback was positive. We did our best to avoid interviewing only participants who were more engaged with the interventions and, therefore, more likely to have favorable views, by purposively sampling participants based on a range of engagement levels. However, a limitation of this study is that we were not able to interview any participants who did not respond to 6-month follow-up in the wider iDEAS trial. Those responding at 6 months may have been more engaged with the app and the trial than those who did not respond at 6-month follow-up and, therefore, may have had more positive views of the digital interventions than those that were less engaged. In a similar vein, is possible that participants who were successful in their alcohol reduction goals would have held more positive views on the intervention. We did not measure whether participants felt they had achieved their alcohol reduction goals, and as such, this is something we cannot examine.

### Conclusions

The Drink Less app appears to be an acceptable intervention for increasing- and higher-risk drinkers captured in this sample. To further this work, future research could examine the relationship between acceptability and engagement and consider how engagement with the app can be increased in the longer term. The NHS alcohol advice web page was not considered acceptable as a stand-alone intervention but may act as a positive signpost for some users.

## Appendix

Multimedia Appendix 1 Interview schedule.

Multimedia Appendix 2 COREQ (Consolidated Criteria for Reporting Qualitative Research) checklist.

Multimedia Appendix 3 Public and patient involvement feedback on results.

## Abbreviations

COREQ: Consolidated Criteria for Reporting Qualitative Research
NHS: National Health Service
TFA: Theoretical Framework of Acceptability
UCL: University College London

## Data Availability

The data sets generated and analyzed during this study are available from the corresponding author on reasonable request.
