# Supplementary material for: Evaluating the Acceptability of the Drink Less App and the National Health Service Alcohol Advice Web Page: Qualitative Interview Process Evaluation
Source: J Med Internet Res. 2024 Jul 18;26:e42319. doi: 10.2196/42319 (PMC11294780; doi:10.2196/42319)
Supplement: Multimedia Appendix 1 [file jmir_v26i1e42319_app1.docx]

**Multimedia Appendix 1 - Interview Schedule**

Throughout this interview we will be asking you about your experience of using the digital support, in your case this was the [*Drink Less* app/ NHS Alcohol advice webpage, delete as needed]. We would like you to make a distinction between your use of the support, and your experience of taking part in the trial (so this would be the follow up surveys and any contact you had with us). The next questions are strictly focused on what you thought about the *Drink Less* app/NHS Alcohol advice webpage and your experience of using it.

1. When you originally signed up to the iDEAS trial in [month], why did you decide to take part?
2. Can you tell me about your experience of using the *Drink Less* app/NHS Alcohol advice webpage?
3. In what situations did you use the *Drink Less* app/NHS Alcohol advice webpage and why?
4. How would you rate the *Drink Less* app/NHS Alcohol advice webpage on a scale of 1 to 5 stars? And can you tell me why? [Global acceptability]
5. How much did you like the *Drink Less* app/NHS Alcohol advice webpage? [Affective Attitude]
6. Do you believe the *Drink Less* app/NHS Alcohol advice webpage was suited to your individual needs? [Perceived Personal Relevance]
7. Do you believe that using the *Drink Less* app/NHS Alcohol advice webpage helped you to drink less? [Perceived personal usefulness/ perceived effectiveness]
8. Did you find the *Drink Less* app/NHS Alcohol advice webpage time-consuming? [Burden]
9. Did you have any other difficulties using it? [Burden/Intervention Coherence]
10. Do you think anyone could use this app? [Ethicality]
11. Did using the *Drink Less* app/NHS Alcohol advice webpage interfere with anything else important to you? [Opportunity costs]
12. How confident were you about using the *Drink Less* app/NHS Alcohol advice webpage? [Self-efficacy]
13. Was it clear to you how to use the *Drink Less* app/NHS Alcohol advice webpage and how it worked? [Intervention coherence]
14. Do you have any other comments you would like to make?

Now, these final three questions refer to the trial - to the follow up surveys and any contact you had with the researchers.

1. How did you find taking part in the wider trial?
2. How would you rate taking part in the trial on a scale of 1 to 5 stars? And can you tell me why? [Global acceptability]
3. Did the financial incentives encourage you to take part in the study?
4. [If yes], to what extent did the financial incentives for the study encourage you to take part?
