# Supplementary material for: Evaluating the Acceptability of the Drink Less App and the National Health Service Alcohol Advice Web Page: Qualitative Interview Process Evaluation
Source: J Med Internet Res. 2024 Jul 18;26:e42319. doi: 10.2196/42319 (PMC11294780; doi:10.2196/42319)
Supplement: Multimedia Appendix 3 [file jmir_v26i1e42319_app3.docx]

**Multimedia Appendix 3 - PPI Feedback on Results**

NHS Alcohol Advice Webpage

Participant 1

**How well does this results section summarise your experience of using the NHS Alcohol advice webpage? Does it capture your thoughts and views on the acceptability of the NHS Alcohol advice webpage?**

I do agree with the affective attitude section about the NHS Alcohol advice webpage (in fact, I think the patronising comment was from me!). From what I recall there wasn’t many positive reasons outlined on the website about cutting down on drinking so it was all outlined in a negative light. It didn’t update or change so it was a ‘once you’ve read it, you feel like you’re repeating’ feel to it.

The ease of access for the website was excellent, and I think as a resource it was clear enough. I imagine they can roll it out into an app easy enough. I wouldn’t visit it for casual reading, I would need to be actively seeking out information about drinking.

I do agree there is always a nervousness about accurately reporting drinking levels, particularly with the stresses that the NHS is under, it makes it seem like it would be even more of a burden to seek help, taking up resources which are needed elsewhere.

The comments are very accurate about the perceived personal relevance – it would have been useful to be able to tailor the offering, but this would likely be difficult and expensive. It also needs to be generic for the overall population.

**Do you think there are key points that are missing from this summary?**

Not that I can remember – maybe something about how it did impact on people’s drinking? I do agree with the comments about willpower.

**Do you have any other comments?**

I thought the trial and website were useful in the sense of making me think more about my drinking, however, this was down to the trial as opposed to the website itself.

There wasn’t really anything about how easy the site is to find – I think I mentioned (I can’t remember) that we had direct links to the NHS site but I don’t know how easy it would be to find the right page if it was from ‘standing’ if I wanted to find the info.

Participant 2

**How well does this results section summarise your experience of using the NHS Alcohol advice webpage? Does it capture your thoughts and views on the acceptability of the NHS Alcohol advice webpage?**

I was quite relieved to find others had the same experience of the website as me, so yes it does reflect my experience. Most of the responses were very much the way I was thinking about the website and where there were differences I can easily see how people might have reached that response. So overall a pretty good reflection of my experience.

**Do you think there are key points that are missing from this summary?**

I think all the key points are covered including a few that hadn’t occurred to me but which seem relevant.

**Do you have any other comments?**

The only interesting thing to add from my point of view would have been a revisit of the respondees after a longer interval. As although I did not find the NHS Alcohol advice webpage much help at the time, in the long term I have substantially cut back on alcohol consumption since and maybe some of the advice did have an influence?

Participant 3

**How well does this results section summarise your experience of using the NHS**

**website? Does it capture your thoughts and views on the acceptability of the NHS**

**Website?**

I think it has captured my thoughts well. I can see my views represented in comments I am likely to have made and in the views shared by other people. In particular the sparseness of information in the site and they way it doesn't differentiate between different levels of addiction or problematic drinking, and in the fear of consequences.

**Do you think there are key points that are missing from this summary?**

Taking on board other people's comments about how NHS is a trusted website and the go to place for medical information, it is really a missed opportunity of it doesn't support everyone in the way they need. In particular in educating, giving case studies, demonstrating the progression of the illness, and rebranding early stage addiction to ensure people feel safe, comfortable and motivated to seek help as soon as possible. The earlier you catch it the less destructive it is and more reversible.

**Do you have any other comments?**

Thank you for taking all these comments forward in your research. Hopefully this website will become a more powerful tool to support people with alcohol related issues.

Also, more support for family and friends would be great. Often their well meaning actions can be counter productive and they need moral support as well.

Participant 4

**How well does this results section summarise your experience of using the NHS Alcohol advice webpage? Does it capture your thoughts and views on the acceptability of the NHS Alcohol advice webpage?**

I would agree with the majority of what was said and the views expressed were mostly similar to mine. There’s always a difficulty in making advice relevant to people with different needs, but I think the Website is good as a starting point for anyone wanting to cut back on their drinking. Although there wasn’t anything particularly new that I hadn’t already heard of or read about, the suggestions the Website make are straightforward and do-able. Also the vast majority of people see the NHS as a reliable source of information and so it is the ideal place to start looking for help. I think the list of support services/websites for more serious drinkers is good, as it shows the variety of help available for people when they may feel most alone.

**Do you think there are key points that are missing from this summary?**

From my point of view the summary would broadly reflect my views and so I don’t think there are any major points that are missing.

**Do you have any other comments?**

This survey was a good opportunity to both look at the NHS site, which I probably wouldn’t otherwise have accessed, and to assess my own drinking habits, so it was a positive experience overall.

Drink Less

Participant 5

**How well does this results section this summarises your experience of using the app? Does it capture your thoughts and views on the acceptability of the Drink Less app?**

I feel that in the main, the results summarise my experience of using the app. In particular, using it definitely made me more mindful of my drinking habits and helped me to see how easy it is to go over the recommended weekly units. Also, it helped me to realise how my sleep is affected by alcohol. I agree that the app was easy to use and I liked the praise it gave on green days, but also felt it was non judgemental and encouraging when I had a bad day.

**Do you think there are key points that are missing from this summary?**

I can’t think of anything that’s missing from the summary.

**Do you have any other comments?**

Using the app has definitely made me more mindful of my drinking habits. These days I drink less - particularly because I’m more aware of how alcohol affects my sleep. I do occasionally go over the recommended units, but not very often. I would definitely use the app again and would encourage my husband to use it as well.

Participant 6

**How well does this results section this summarises your experience of using the app? Does it capture your thoughts and views on the acceptability of the Drink Less app?**

Affective attitude: Yes agree.. However, the summary should indicate the exception of cases where people were a little discouraged when they were unable to succeed in their goals week after week. This was certainly true of me on some weeks

Burden: Yes it was not burdensome to use the app. Easy and quick

Ethicality: Yes agree app was accessible

Intervention Coherence: Yes app was easy to use

Opportunity Costs: Yes agree.. fact that it was easy to backfill for the week was helpful

Perceived Effectiveness: Yes agree.. it was effective certainly for me during the weeks when i used it.. however not sure what would happen over longer periods of time.

Perceived Personal Relevance: Makes sense after reading the summary but does not think it applied to me

Perceived Self Efficacy: Yes agree.. concern for me would be the ability to use for a long period of time. Unsure if that would be possible

Some of the labels for the summaries are confusing for me i.e., ethicality, intervention cohorerence

**Do you think there are key points that are missing from this summary?**

I think it would be good to include stats around how many people were able to use the app over long periods of time. Also if you have data on whether once they stopped using the app how long they reduced drinking or whether they fell back into old habits.

**Do you have any other comments?**

One suggestion for the app that I forgot to provide before was: It would be nice to link people into helplines or even paid professionals who can help with drinking. I think this would be nice addition to the app as if you feel like you are failing and feel helpless there is an avenue to get more help

Participant 7

**How well does this results section summarise your experience of using the app? Does it capture your thoughts and views on the acceptability of the Drink Less app?**

Very well indeed. Not all necessarily my experience, but understand what people are getting at in the comments.

**Do you think there are key points that are missing from this summary?**

No – not from my experience of using the app

**Do you have any other comments?**

None - thanks

Participant 8

**How well does this results section summarise your experience of using the app? Does it capture your thoughts and views on the acceptability of the Drink Less app?**

Generally the comments summarise my experience. In particular issues in relation to:

- The app helps you only if you are motivated.
- It would benefit from a human touch, such as weekly zoom calls.
- The range of tools can be overwhelming in the beginning.
- You stop using it while you are on holiday.
- Seeing all the green days is great.
- It’s great for tracking how much you drink and compare it against the rest of the population.

**Do you think there are key points that are missing from this summary?**

The way it compares my drinking against the rest of the population is very interesting. I found it shocking in the beginning but now I’m wondering if it is exaggerating bit.

**Do you have any other comments?**

I started using this app a couple of years ago when I had a lot of motivation. I stopped drinking for several months and the app really helped. Especially the if… then… method. However, now I’m drinking again and the app cannot help me. Maybe I need some face to face support. Thank you very much for developing this app. It was very helpful for me when I was feeling unsupported and lost. Keep up the good work!
